# Prominin‐1 expression in the testis/epididymis and fertility

**DOI:** 10.1002/rmb2.12544

**Published:** 2023-10-04

**Authors:** Christine A. Fargeas, József Jászai, Denis Corbeil

**Affiliations:** ^1^ Tissue Engineering Laboratories, Biotechnology Center (BIOTEC) Center for Molecular and Cellular Bioengineering and Medizinische Fakultät der Technischen Universität Dresden Dresden Germany; ^2^ Institute of Anatomy Medizinische Fakultät der Technischen Universität Dresden Dresden Germany

**Keywords:** CD133, prominin‐1, reproductive system, sperm cell

## Abstract

The contribution of Prominin‐1 (aka CD133) to male fertility has recently been (re)investigated, with contradictory results. Early findings, essential for deciphering its role, have unfortunately been neglected. Here, the authors present what is currently known about its expression in the male reproductive system of rodents and men so that its involvement in male fertility can be re‐examined and discussed in the light of these elements.

## 1 INTRODUCTION

Matsukuma and colleagues have recently reported that *Prominin‐1* deletion results in spermatogenic impairment, sperm morphological defects, and infertility in mice.^1^ Prominin‐1 (alias CD133) has been, for more than two decades, the subject of intensive research in various organ systems due to its expression in stem (cancer stem) cells. This pentaspan transmembrane glycoprotein (≈120 kDa) localizes specifically in highly curved membranes as in microvilli and cilia of diverse epithelial cells and in other types of protrusions of non‐epithelial cells. It regulates the organization and/or dynamics of such membrane protrusions via its interactions with membrane lipids and cytoplasmic proteins.

## 2 EXPRESSION OF PROMININ‐1 IN MALE REPRODUCTIVE SYSTEMS

We reported, in 2004, its expression in murine testes and epididymides.^2^ Using immunohistochemistry and electron microscopy, we demonstrated that Prominin‐1 was concentrated in the stereocilia of the apical surface of principal cells lining the epididymal epithelium, all along the duct with the exception of the initial segment (see Table 1). Prominin‐1 was also found on the tails of developing spermatozoa associated with contorted testis seminiferous tubules, suggesting that this molecule may play a role in spermiogenesis (the final stage of spermatogenesis), in line with its preferential subcellular localization in membrane protrusions.^2^ Several splice variants of Prominin‐1 with alternative C‐termini were characterized, exhibiting lower molecular weights and differential glycosylation compared with the 120‐kDa kidney form originally identified. This means that, depending on the antibody used, the full spectrum of Prominin‐1 variants may not be detected, notably in the reproductive system (Table 1).^2^ Prominin‐1 variants with different molecular weights are indeed differentially expressed in *ductuli efferentes* and the epididymal tract,^2^ and only a 100‐kDa form of Prominin‐1 harboring a truncated cytoplasmic C‐terminus appears in the testis.^2^, ^3^ The relation of these alternative C‐termini with differential function of Prominin‐1 in these tissues remains to be experimentally addressed. *Prominin‐1* transcripts were later mapped in the luminal zone of the contorted seminiferous tubules containing spermatids and in the epididymal duct by in situ hybridization (Table 1).^4^ Matsukuma and colleagues confirmed this last observation by β‐galactosidase staining of heterozygote animals of a knock‐in mouse model carrying the *LacZ* gene in the *Prom1* locus.^1^ Strikingly, while neither *Prominin‐1* transcript expression^4^ nor LacZ staining^1^ were detected in the basal portion of seminiferous tubules containing actively dividing spermatogonia, the immunodetection in the latter study contradicted these findings and also failed to detect Prominin‐1 protein in developing spermatozoa.^1^ The reason for these discrepancies was not discussed by the authors and remains to be determined. The use of an anti‐Prominin‐1 antibody (HPA004922; Sigma‐Aldrich), which is directed against a sequence of human Prominin‐1 sharing only 56% identity with its mouse counterpart, may be part of the answer. Actually, most anti‐Prominin‐1 antibodies do not cross‐react between mouse and human counterparts.^5^ As this antibody is not reported to react with species other than human, its cross‐reactivity with murine Prominin‐1 needs to be demonstrated first.^1^ As for the antibody (18470‐1‐AP, Proteintech) used for the immunoblot of testicular samples,^1^ although presented as reactive against mouse Prominin‐1 despite being also raised against human Prominin‐1, it is specifically directed against the C‐terminal region of the 120‐kDa kidney form that is partly absent in the 100‐kDa variant expressed in the testis (Table 1). It would therefore be advisable to run samples (kidney and testis) in parallel and probe them with two different antibodies to identify the variants expressed.

**TABLE 1:** Detection of Prominin‐1 in testis and epididymis.

Species			Mouse								Rat	Human	
Study reference			^2^		^3^	^6^	^1^			^4^	^7^	^8^	^9^
	Detection		13A4^a^	αI3^b^	13A4	eBioscience^c^	18 470‐1‐AP^d^	HPA004922^e^	β‐Gal^f^	ISH^g^	18 470‐1‐AP	ABGENT^h^	Miltenyi^i^
Testis	WB		100 kDa	–	+^j^		>4 bands^k^						120 kDa^l^
		(N‐deglycosylated)	(89 kDa)	–	+								
	HC	Spermatogonia	–					(+)^m^		–		sporadic	
		Spermatocytes	–					+		–			
		Spermatids	–						+	+		(+)^m^	
		Spermatozoa	+							–		–	+
		Sertoli cells	–							–			+
		Leydig cells/Macrophage	–							–			
Epididymis	WB		104–112 kDa			100 kDa (Cauda sperm)					112 and 120 kDa		
		(N‐deglycosylated)	(89 kDa)	–									
		(N‐deglycosylated)	(92 kDa)	(92 kDa)									
	HC	Epithelium							+			+	
		Ductuli efferent	+	+						+	+		
		Initial segment	–	–						–	scarce/−		
		Distal caput	+	+							+		
		Corpus	+	–						+ & –	+		
		Cauda	+	+							+		
		Spermatozoa	−	−		faint					faint/−		

*Note*: +, presence of Prominin‐1; −, absence of Prominin‐1; grayed, not applicable or not determined.

Abbreviations: HC, histochemistry; WB, western blot.

^a^ 13A4, rat monoclonal antibody (mAb) targeting the second extracellular domain of mouse Prominin‐1, recognizes all splice variants in testis/epididymis.

^b^ αI3, rabbit antiserum targeting the C‐terminal domain of mouse Prominin‐1.s1, recognizes only non‐truncated variants.

^c^ Antiserum against Prominin‐1 from eBioscience, not specified.

^d^ 18 470‐1‐AP from Proteintech, antigen‐purified rabbit polyclonal targeting the C‐terminus (amino acid 806–856) of human Prominin‐1.s1, reacts with human, mouse, and rat; recognition of splice variants affecting the C‐terminal domain has not been validated.

^e^ HPA004922 from Sigma‐Aldrich, antigen‐purified rabbit polyclonal targeting sequence in the 2nd extracellular domain (amino acid 209–315) of human Prominin‐1.s1, reacts with human, not validated for mouse.

^f^ β‐Galactosidase staining on *Prom1* ^*+/lacZ,DTA*^.

^g^ ISH, in situ hybridization of *Prominin‐1* transcript.

^h^ Mouse anti‐PROM1 mAb from ABGENT, not specified.

^i^ Anti‐CD133 mAb from Miltenyi Biotec, not specified.

^j^ Molecular weights not precisely determined, but consistent with those of Ref. 2 using the same antibody.

^k^ 4 or more immunoreactive bands up to 150 kDa present in *Prom1* ^+/+^ and *Prom1* ^−/−^ testis.

^l^ Results obtained on micro‐testicular sperm extraction of obstructive azoospermia patients show additional bands of lower molecular weight.

^m^ Parentheses indicate elements visible on the figure, but not mentioned as such by the authors.

Mammalian spermatozoa gain fertility potential during their transit through the epididymis. In such context, Prominin‐1 may participate in sperm maturation, either by the generation of small vesicles conveying maturation factors from the epididymis, as seminal fluid does contain Prominin‐1^+^ extracellular vesicles, or through its shedding from spermatozoa with other sperm constituents. Indeed, Asano and colleagues suggested that murine sperm cells acquired Prominin‐1 in the testis, and not gradually during epididymal maturation (Table 1).^6^ Congruently, Prominin‐1 was hardly detected on rat spermatozoa in the luminal compartment of the *ductuli efferentes*, initial segment, and *caput*, and somewhat inconsistently, in the *corpus epididymis*.^7^ In humans, Prominin‐1 was detected in sperm cells, epididymal epithelium,^8^, ^9^ and sporadically in fetal tissues in the second but not the third trimester of gestation (Table 1).^8^ Consistent with its expression in germ cells, upregulation of Prominin‐1 was observed in seminoma. In normal adult testicular tissues, its mRNA expression was nonetheless hardly detectable by PCR.^8^ While evaluating differences in expression profiles of biomarkers between healthy and infertile men, Yukselten and colleagues have shown that Prominin‐1 is expressed in healthy Sertoli cells and sperm cells (Table 1).^9^ How these differences between species are related to the expression of distinct Prominin‐1 variants, to differential regulation or simply reflect a constitutive interspecific divergence might be worth exploring.

## 3 DIFFERENT MURINE MODELS OF PROMININ‐1 DEFICIENCY

Matsukuma and colleagues also report that deletion of *Prominin‐1* results in complete infertility in male mice,^1^ which seems to contradict other reports of diverse homozygous *Prominin‐1*‐deficient mouse models being viable and fertile (Table 2). These animal models show various retinal degeneration phenotypes, but no apparent fertility problems. Of note, Karim and colleagues were the first to observe compromised spermatogenesis in some (without indicating in which proportion) *Prom1* ^*−/*−^ males on a less characterized 129SvEv background, although they reported no interference with development or fertility in general.^10^ It appears therefore that the drastic effect on male fertility reported by Matsukuma and colleagues may be influenced by factors other than the sole absence of Prominin‐1.^1^ One cannot exclude that a substrain of *Prom1* ^*−/*−^ may have been derived given that the original publication in 2009 of these engineered animals did not report fertility issues and the distributor (Strain Data Sheet, BRC No. RBRC05284; RIKEN BRC, Japan) describes homozygous mice as viable and fertile. Difficulties in breeding on the C57BL6 background were reported in 2015 and remedied by generating a hybrid C57BL/6xCBA/NSlc knockout through in vitro fertilization (Table 2). The differences in phenotype of the different knockout models, whether due to environmental factors or related to the strains and backgrounds of the mice, call for more detailed studies with established tools and larger cohorts of samples, to clarify the involvement of Prominin‐1 in spermatogenesis. In particular, it could be interesting to see whether the other *Prominin‐1*‐deficient mouse models develop in a time course study the same tendency to higher sperm defect, to pinpoint the role of Prominin‐1 in male fertility. Finally, it is noteworthy that, although numerous recessive and dominant mutations in human *PROM1* gene were reported to cause retinal degeneration, no other major phenotype, including infertility, was, to our knowledge, documented in these patients.

**TABLE 2:** *Prom1*‐ablated murine models.

Genotype	Disrupting exonGene ID 19126	Type	Background	Reproductive phenotype	Link
*Prom1* ^*−/−*^	Exon 2	Constitutive knockout	congenic C57BL/6	Healthy and fertile	PMID:19228982
			50/50 129/swiss	–	PMID:21337465
			congenic C57BL/6JOlaHsd	Viable and fertile	PMID:23509298
*Prom1* ^*lacZ,DTA/lacZ,DTA*^	Exon 2	LacZ knockin	C57BL/6	Viable and develop normally. No indication of fertility problem	PMID:19718438
				Homozygous mice are viable and fertile	https://knowledge.brc.riken.jp/resource/animal/card?__lang__=en&brc_no=RBRC05284
			C57BL/6	Difficulty in breeding	PMID:25414197
			C57BL/6xCBA/NSlc (Hybrid obtained by in vitro fertilization)	–	
*Prom1* ^*C‐L/C‐L*^	Exon 2	CreER LacZ knockin	C57BL/6	Born and aged normally. No mention regarding fertility	PMID:19092805
*Prom1* ^*−/−*^	?	?	129vEv	No interference with development or fertility, but compromised spermatogenesis in some *Prom1* ^*−/−*^ males Moderate degree of germinal arrest with multifocal seminiferous tubule atrophy and degeneration and arrest of spermatogenesis at 4 months of age	PMID:25452936
*Prom1* ^*−/−*^	Exon 2	Constitutive knockout/CRISPR/Cas 9 *Prom1* ^*−/−*^	C57BL/6	No apparent abnormal phenotype, healthy and fertile	PMID:34540608
*Prom1* ^*rd19/rd19*^ B6.BXD83‐Prom1^rd19^/BocJ	Exon 9	Spontaneous null knockout (premature stop codon; K269X)	C57BL/6J	Homozygous null mice develop and reproduce normally	MGI: J:5605699 PMID:30328220

*Note*: ?, information not provided.

Abbreviations: MGI, mouse genome informatics (see PMID: 33231642); PMID, PubMed identifier.

## CONFLICT OF INTEREST STATEMENT

The authors declare that the research was conducted in the absence of any commercial or financial relationships that could be construed as a potential conflict of interest.

## ANIMAL RIGHTS

No animal experiments have been performed in this article.
